# Another operator-theoretical proof for the second-order phase transition in the BCS-Bogoliubov model of superconductivity

**DOI:** 10.1038/s41598-022-11652-4

**Published:** 2022-05-19

**Authors:** Shuji Watanabe

**Affiliations:** Division of Mathematical Sciences, Graduate School of Engineering, Gunma University, 4-2 Aramaki-machi, Maebashi, 371-8510 Japan

**Keywords:** Applied mathematics, Superconducting properties and materials

## Abstract

In the preceding papers, imposing certain complicated and strong conditions, the present author showed that the solution to the BCS-Bogoliubov gap equation in superconductivity is twice differentiable only on the neighborhoods of absolute zero temperature and the transition temperature so as to show that the phase transition is of the second order from the viewpoint of operator theory. Instead, we impose a certain simple and weak condition in this paper, and show that there is a unique nonnegative solution and that the solution is indeed twice differentiable on a closed interval from a certain positive temperature to the transition temperature as well as pointing out several properties of the solution. We then give another operator-theoretical proof for the second-order phase transition in the BCS-Bogoliubov model. Since the thermodynamic potential has the squared solution in its form, we deal with the squared BCS-Bogoliubov gap equation. Here, the potential in the BCS-Bogoliubov gap equation is a function and need not be a constant.

## Introduction

In the BCS-Bogoliubov model of superconductivity, one does not show that the solution to the BCS-Bogoliubov gap equation is partially differentiable with respect to the absolute temperature *T*. Nevertheless, without such a proof, one partially differentiates the solution and the thermodynamic potential with respect to the temperature twice so as to obtain the entropy and the specific heat at constant volume. One then shows that the phase transition from a normal conducting state to a superconducting state is of the second order. Therefore, if the solution were not partially differentiable with respect to the temperature, then one could not partially differentiate the solution and the thermodynamic potential with respect to the temperature, and hence one could not obtain the entropy and the specific heat at constant volume. As a result, one could not show that the phase transition is of the second order. For this reason, it is highly desirable to show that there is a unique solution to the BCS-Bogoliubov gap equation and that the solution is partially differentiable with respect to the temperature twice.

In the preceding papers (see^1^[Theorems 2.2 and 2.10] and^2^[Theorems 2.3 and 2.4]), the present author gave a proof of the existence and uniqueness of the solution and showed that the solution is indeed partially differentiable with respect to the temperature twice on the basis of fixed-point theorems. In this way, the present author gave an operator-theoretical proof of the statement that the phase transition is of the second order, and thus solved the long-standing problem of the second-order phase transition from the viewpoint of operator theory. Here, the potential in the BCS-Bogoliubov gap equation is a function and need not be a constant.

But the present author imposed certain complicated and strong conditions in the preceding papers^1–3^. As a result, the present author showed that the solution to the BCS-Bogoliubov gap equation is partially differentiable with respect to the temperature only on the neighborhoods of absolute zero temperature $T=0$ and the transition temperature $T=T_c$. Instead, we impose a certain simple and weak condition in this paper. Thanks to this simple and weak condition, we show that there is a unique nonnegative solution and that the solution is indeed partially twice differentiable with respect to the temperature on the interval $[T_0, \, T_c]$ as well as pointing out several properties of the solution. Here, the temperature $T_0$ is defined in (1.4) below, and the temperature interval $[T_0,\, T_c]$ can be nearly equal to the whole temperature interval $[0,\, T_c]$ (see Remark 2.1 below).

Differentiating the thermodynamic potential with respect to the temperature, we thus give another operator-theoretical proof for the second-order phase transition. As is well known, the thermodynamic potential has the squared solution in its form, not the solution itself. Therefore, we deal with the squared BCS-Bogoliubov gap equation, not the BCS-Bogoliubov gap equation. From the viewpoint of operator theory, the present author thinks that dealing with the squared BCS-Bogoliubov gap equation provides a straightforward way to show the second-order phase transition.

The BCS-Bogoliubov gap equation^4,5^ is a nonlinear integral equation given by

1.1 $$\begin{aligned} u_0(T,\,x)=\int _I \frac{U(x,\,\xi )\, u_0(T,\, \xi )}{\,\sqrt{\,\xi ^2+u_0(T,\, \xi )^2\,}\,}\, \tanh \frac{\,\sqrt{\,\xi ^2+u_0(T,\, \xi )^2\,}\,}{2T}\, d\xi , \ T \ge 0, \ (x, \,\xi ) \in I^2. \end{aligned}$$

Here, the solution $u_0$ is a function of the absolute temperature *T* (of a superconductor) and the energy *x* (of an electron). The closed interval *I* is given by $I=[\varepsilon ,\, \hslash \omega _D]$, where the Debye angular frequency $\omega _D$ is a positive constant and depends on a superconductor, and $\varepsilon >0$ is a cutoff (see the following remark). The potential $U(\cdot ,\,\cdot )$ satisfies $U(x,\,\xi )>0$ at all $(x,\,\xi ) \in I^2$. Throughout this paper we use the unit where the Boltzmann constant $k_B$ is equal to 1.

### Remark 1.1

For simplicity, we introduce the cutoff $\varepsilon >0$ in (1.1). Here, the cutoff $\varepsilon >0$ is small enough. We see that the cutoff is unphysical, but we introduce it for simplicity.

In^1–3,6–22^, the existence, the uniqueness and several properties of the solution to the BCS-Bogoliubov gap equation were established and studied. See also Kuzemsky^23^[Chapters 26 and29] and^24,25^. Anghel and Nemnes^26^ and Anghel^27,28^ showed that if the physical quantity $\mu$ in the BCS-Bogoliubov model is not equal to the chemical potential, then the phase transition from a normal conducting state to a superconducting state is of the first order under a certain condition without any external magnetic field. Introducing imaginary magnetic field, Kashima^29–32^ pointed out that the phase transition is of the second-order if and only if a certain value is greater than $\sqrt{17-12\sqrt{2}}$ and that the phase transition is of order $4n+2$ if and only if the value above is less than or equal to $\sqrt{17-12\sqrt{2}}$. Here. *n* is an arbitrary positive integer.

In this connection, the BCS-Bogoliubov gap equation in superconductivity plays a role similar to that of the Maskawa–Nakajima equation^33,34^ in elementary particle physics. In Professor Maskawa’s Nobel lecture, he stated the reason why he considered the Maskawa-Nakajima equation. See the present author’s paper^35^ for an operator-theoretical treatment of the Maskawa–Nakajima equation.

Squaring both sides of the BCS-Bogoliubov gap equation and putting $f_0(T,\,x)=u_0(T,\,x)^2$ give the squared BCS-Bogoliubov gap equation:

$$\begin{aligned} f_0(T,\,x)=\left( \int _I U(x,\,\xi ) \sqrt{ \, \frac{f_0(T,\, \xi )}{\, \xi ^2+f_0(T,\, \xi )\,} \,} \, \tanh \frac{\,\sqrt{\,\xi ^2+f_0(T,\, \xi )\,}\,}{2T}\, d\xi \right) ^2. \end{aligned}$$

Let $T_c$ be the transition temperature (see^20^[Definition 2.5] for our operator-theoretical definition of $T_c$) and let $D=[T_0,\, T_c] \times I \in {\mathbb {R}}^2$. Here, $I=[\varepsilon ,\, \hslash \omega _D]$. Define our operator *A* by

1.2 $$\begin{aligned} Af(T,\,x)=\left( \int _I U(x,\,\xi ) \sqrt{ \, \frac{f(T,\, \xi )}{\, \xi ^2+f(T,\, \xi )\,} \,} \, \tanh \frac{\,\sqrt{\,\xi ^2+f(T,\, \xi )\,}\,}{2T}\, d\xi \right) ^2, \quad (T,\,x) \in D, \end{aligned}$$

where $f \in W$ (see (2.2) below for the subset *W*). We define our operator *A* on the subset *W* and look for a fixed point of our operator *A*. Note that a fixed point of *A* becomes a solution to the squared BCS-Bogoliubov gap equation, and that its square root becomes a solution to the BCS-Bogoliubov gap equation (1.1).

Let $U_1$ and $U_2$ be positive constants, where $(0<) \, U_1 \le U_2$. If the potential $U(\cdot ,\,\cdot )$ is a positive constant and $U(x,\,\xi )=U_1$ at all $(x,\,\xi ) \in I^2$, then the solution to the BCS-Bogoliubov gap equation (1.1) becomes a function of the temperature *T* only. Denoting the solution by $T \mapsto \Delta _1(T)$, we have (see^4^)

1.3 $$\begin{aligned} 1=U_1 \int _I \frac{1}{\,\sqrt{\,\xi ^2+\Delta _1(T)^2\,}\,} \, \tanh \frac{\, \sqrt{\,\xi ^2+\Delta _1(T)^2\,}\,}{2T}\,d\xi , \quad 0 \le T \le \tau _1. \end{aligned}$$

Here, the temperature $\tau _1>0$ is defined by (see^4^)

$$\begin{aligned} 1=U_1 \int _I \frac{1}{\,\xi \,} \, \tanh \frac{\xi }{\,2\tau _1\,}\,d\xi . \end{aligned}$$

The solution $T \mapsto \Delta _1(T)$ is continuous and strictly decreasing with respect to *T*, and moreover, the solution is of class $C^2$ with respect to *T*. For more details, see^20^ [Proposition 1.2].

We set $\Delta _1(T)=0$ at all $T \ge \tau _1$. Then (1.3) becomes

$$\begin{aligned} 1>U_1 \int _I \frac{1}{\,\xi \,} \, \tanh \frac{\xi }{\,2T \,}\,d\xi , \quad T>\tau _1. \end{aligned}$$

We choose an arbitrary temperature $T_0 \; (>\tau _1)$. Then, for $T \in [T_0,\, T_c]$,

1.4 $$\begin{aligned} U_1 \int _I \frac{1}{\,\xi \,} \, \tanh \frac{\xi }{\,2T \,}\,d\xi <1. \end{aligned}$$

On the other hand, If $U(x,\,\xi )=U_2$ at all $(x,\,\xi ) \in I^2$, then we have the solution $T \mapsto \Delta _2(T)$ to

1.5 $$\begin{aligned} 1=U_2 \int _I \frac{1}{\,\sqrt{\,\xi ^2+\Delta _2(T)^2\,}\,} \, \tanh \frac{\, \sqrt{\,\xi ^2+\Delta _2(T)^2\,}\,}{2T}\,d\xi , \quad 0 \le T \le \tau _2. \end{aligned}$$

Here, the temperature $\tau _2>0$ is defined by

$$\begin{aligned} 1=U_2 \int _I \frac{1}{\,\xi \,} \, \tanh \frac{\xi }{\,2\tau _2\,}\,d\xi . \end{aligned}$$

The solution $T \mapsto \Delta _2(T)$ has properties similar to those of the solution $T \mapsto \Delta _1(T)$. We again set $\Delta _2(T)=0$ at all $T \ge \tau _2$.

The inequality $U_1 \le U_2$ implies

$$\begin{aligned} \Delta _1(T) \le \Delta _2(T) \quad (0 \le T \le \tau _2). \end{aligned}$$

For the graphs of $\Delta _1(\cdot )$ and $\Delta _2(\cdot )$, see^2^[Figure 1].

## Main results

Suppose that the potential $U(\cdot ,\,\cdot )$ in the BCS-Bogoliubov gap equation (1.1) satisfies the following conditions:

2.1 $$\begin{aligned} U(\cdot ,\,\cdot ) \in C^2(I^2), \quad (0<) \, U_1 \le U(x,\,\xi ) \le U_2 \quad \hbox {at all}\quad (x,\,\xi ) \in I^2, \end{aligned}$$

and (2.3) below.

The inequalities $U_1 \le U(x,\,\xi ) \le U_2$ at all $(x,\,\xi ) \in I^2$ imply $\tau _1 \le T_c \le \tau _2$ (see^20^[Remark 2.6]). Set

$$\begin{aligned} a=\left\{ \frac{ \displaystyle { \max _{(x,\,\xi ) \in I^2} U(x,\,\xi ) }}{\, \displaystyle { \min _{(x,\,\xi ) \in I^2} U(x,\,\xi ) } \,} \right\} ^2 \ (\ge 1). \end{aligned}$$

### Remark 2.1

The temperatures $\tau _1$, $T_0$, $T_c$, $\tau _2$ satisfy $(0<)\, \tau _1<T_0<T_c \le \tau _2$. If $U_1$ is small enough, then so is $\tau _1$. Therefore, $T_0$ can be small enough, and hence $T_0$ does not need to be close to the transition temperature $T_c$. As a result, the temperature interval $[T_0,\, T_c]$ can be nearly equal to the whole temperature interval $[0,\, T_c]$. For temperatures at or near zero temperature, the smoothness of the solution to the BCS-Bogoliubov gap equation with respect to such temperatures has been shown in^1^[Theorem 2.2]. In this paper we thus deal with the temperature interval $[T_0,\, T_c]$.

Let *W* be a subset of the Banach space *C*(*D*) satisfying

2.2 $$\begin{aligned} W= & {} \big \{ f \in C^2(D) (\subset C(D)) : (0 \le ) \ \Delta _1(T)^2 \le f(T,\,x) \le \Delta _2(T)^2, \quad f(T_c,\,x) = 0, \\ \nonumber&\frac{f(T,\,x)}{\, f(T,\,x_1) \,} \le a, \quad -f_T(T,\,x)>0, \quad \max _{(T,\, x) \in D} \left\{ -f_T(T,\,x) \right\} \le M_T \big \}. \end{aligned}$$

Here, the norm of the Banach space *C*(*D*) is given by

$$\begin{aligned} \Vert g \Vert =\sup _{(T,\, \xi ) \in D} | \, g(T,\, \xi ) \, |, \quad g \in C(D), \end{aligned}$$

and

$$\begin{aligned} M_T=\frac{4a \, U_2 \, \displaystyle { \left( \max _{z \ge 0} \frac{z}{\, \cosh z \,} \right) ^2 } }{ \, \varepsilon \, U_1 \, \displaystyle { \left( \tanh \frac{\varepsilon }{\, 2T_c \,}-\frac{\varepsilon }{\, 2T_c \,} \frac{1}{\cosh ^2 \frac{\varepsilon }{\, 2T_c \,} } \right) \int _I \frac{d\xi }{\, \left( \xi ^2+\Delta _2(0)^2 \right) ^{3/2} \,} \, } } \quad (>0). \end{aligned}$$

Note that

$$\begin{aligned} \sup _{f \in W} \left[ \max _{(T,\, x) \in D} \left\{ -f_T(T,\,x) \right\} \right] = M_T. \end{aligned}$$

### Remark 2.2

The inequality $f(T,\,x) / f(T,\,x_1) \le a$ in the definition of *W* is not defined at $T=T_c$ since $f(T_c,\,x) = 0$. For $T<T_c$, there is a $T_1$ ($T<T_1<T_c$) such that $f(T,\,x)=(T-T_c) \, f_T(T_1,\,x)$. Therefore, $f(T_c,\,x) / f(T_c,\,x_1)$ is defined to be $f_T(T_c,\,x) / f_T(T_c,\,x_1)$.

### Remark 2.3

The conditions imposed in the previous papers of the present author^1^[Condition (C)] and^2^[Condition (C)] were very complicated, and so it was very tough to show the existence, uniqueness and smoothness of the solution to the BCS-Bogoliubov gap equation (1.1). Instead, we impose the simple condition that $f \in C^2(D)$ and $f(T_c,\,x) = 0$ in the definition of the subset *W* (see (2.2)). Thanks to this simple condition, it is straightforward to show the existence, uniqueness and smoothness of the solution.

Let us remind here that for $T \in [T_0,\, T_c]$ (see (1.4)),

$$\begin{aligned} \int _I \frac{\, U_1 \,}{\,\xi \,} \, \tanh \frac{\xi }{\,2T \,}\,d\xi <1. \end{aligned}$$

Note that $a \ge 1$ and that $U(x,\,\xi ) \ge U_1$ at all $(x,\,\xi ) \in I^2$. We then let the potential $U(\cdot ,\,\cdot )$ satisfy

2.3 $$\begin{aligned} a^{1/4} \max _{(T,\, x) \in D} \left[ \, \int _I \frac{\, U(x,\, \xi ) \,}{\xi } \, \tanh \frac{\xi }{\, 2T\,}\,d\xi \, \right] \le 1. \end{aligned}$$

### Remark 2.4

The following two theorems hold true not only when the potential $U(\cdot ,\,\cdot )$ in the BCS-Bogoliubov gap equation (1.1) is a positive constant, but also when $U(\cdot ,\,\cdot )$ is a function. See Remark 3.8 below.

We denote by ${\overline{W}}$ the closure of *W* with respect to the norm $\Vert \cdot \Vert$ mentioned above. The following are our main results.

### Theorem 2.5

Let the potential $U(\cdot ,\,\cdot )$ in the BCS-Bogoliubov gap equation (1.1) satisfy (2.1) and (2.3). Let *W* be as in (2.2). Then there is a unique fixed point $f_0 \in {\overline{W}}$ of our operator $A: \, {\overline{W}} \rightarrow {\overline{W}}$. Therefore, there is a unique nonnegative solution $u_0=\sqrt{f_0}$ to the BCS-Bogoliubov gap equation (1.1).

Let $f_0$ be the fixed point given by Theorem 2.5 in the following two remarks, where several properties of the solution $u_0=\sqrt{f_0}$ to the BCS-Bogoliubov gap equation (1.1) are pointed out. Suppose $f_0 \in W$. If $f_0$ is an accumulating point of *W*, then $f_0$ can be approximated by an element $f \in W$, and the very element $\sqrt{f}$ satisfies the following properties instead of $u_0$.

### Remark 2.6

$u_0 \in C^2([T_0,\, T_1] \times I)$, where $T_1>0$ is arbitrary as long as $T_1<T_c$. Since $f_0(T_c,\, x)=0$ and $(\partial f_0/\partial T)(T,\, x)<0$ at *T* in a neighborhood of $T_c$, it follows that $u_0(T_c,\, x)=0$ and $(\partial u_0/\partial T)(T,\, x)<0$ at *T* in a neighborhood of $T_c$. Moreover, $(\partial u_0/\partial T)(T,\, x)\rightarrow -\infty$ as $T \uparrow T_c$. But $(\partial u_0^2/\partial T)(T,\, x)\rightarrow (\partial f_0/\partial T)(T_c,\, x)$ as $T \uparrow T_c$.

### Remark 2.7

The inequalities $\Delta _1(T) \le u_0(T,\,x) \le \Delta _2(T)$ and $\displaystyle { \frac{\, u_0(T,\, x)\, }{\, u_0(T,\, x_1) \,} \le \sqrt{a}}$ hold.

In order to show that the transition from a normal conducting state to a superconducting state at $T=T_c$ is of the second-order, we need to deal with the thermodynamic potential $\Omega$ and differentiate it with respect to the temperature *T* twice. Note that the thermodynamic potential $\Omega$ has the fixed point $f_0 \in {\overline{W}}$ given by Theorem 2.5 in its form, not the solution $\sqrt{ f_0 }$ to the BCS-Bogoliubov gap equation (1.1) in its form. As mentioned before, this is why we treat the squared BCS-Bogoliubov gap equation, not the equation itself . See^1^[(1.5), (1.6)] and^2^[(1.6)] for the form of the thermodynamic potential $\Omega$. See also^2^[Definition 1.10] for the operator-theoretical definition of the second-order phase transition.

### Theorem 2.8

Let the potential $U(\cdot ,\,\cdot )$ in the BCS-Bogoliubov gap equation (1.1) satisfy (2.1) and (2.3). Let *W* be as in (2.2). Then the transition from a normal conducting state to a superconducting state at $T=T_c$ is of the second-order.

## Proofs of Theorems 2.5 and 2.8

### Lemma 3.1

1. The subset *W* is a bounded and convex subset of the Banach space *C*(*D*).
2. The closure ${\overline{W}}$ is a bounded, closed and convex subset of the Banach space *C*(*D*).

### Proof

(1) Note that the function $T \mapsto \Delta _2(T)^2$ is strictly decreasing (see^20^[Proposition 1.2]). Therefore, *W* is bounded since $f(T,\,x) \le \Delta _2(T)^2 \le \Delta _2(0)^2$ for every $f \in W$. In order to show that *W* is convex, it suffices to show that

$$\begin{aligned} \frac{tf(T,\,x)+(1-t)g(T,\,x)}{\, tf(T,\,x_1)+(1-t)g(T,\,x_1)\,} \le a. \end{aligned}$$

 Here, $t \in [0, \, 1]$ and $f, \, g \in W$. Let $T \not = T_c$. Since $f(T,\,x) \le a f(T,\,x_1)$ and $g(T,\,x) \le a g(T,\,x_1)$, it follows

$$\begin{aligned} \frac{tf(T,\,x)+(1-t)g(T,\,x)}{\, tf(T,\,x_1)+(1-t)g(T,\,x_1)\,} \le \frac{t \, af(T,\,x_1)+(1-t)\, ag(T,\,x_1)}{\, tf(T,\,x_1)+(1-t)g(T,\,x_1)\,}=a. \end{aligned}$$

Next let $T=T_c$. We remind Remark 2.2 here. Then

$$\begin{aligned}&\frac{tf(T_c,\,x)+(1-t)g(T_c,\,x)}{\, tf(T_c,\,x_1)+(1-t)g(T_c,\,x_1)\,}= \frac{tf_T(T_c,\,x)+(1-t)g_T(T_c,\,x)}{\, tf_T(T_c,\,x_1)+(1-t)g_T(T_c,\,x_1)\,} \\\le & {} \frac{t \, af_T(T_c,\,x_1)+(1-t)\, ag_T(T_c,\,x_1)}{\, tf_T(T_c,\,x_1)+(1-t)g_T(T_c,\,x_1)\,}=a. \end{aligned}$$

Therefore, *W* is convex.

(2) We have only to show that ${\overline{W}}$ is convex. Let $f, \, g \in {\overline{W}}$. Then there are $\{ f_n \}, \, \{ g_n \} \subset W$ satisfying $f_n \rightarrow f$ and $g_n \rightarrow g$ in the Banach space *C*(*D*). Since *W* is convex, $tf_n+(1-t)g_n \in W$ for $t \in [0, \, 1]$.

$$\begin{aligned} \left\| \, \{ tf+(1-t)g \}- \{ tf_n+(1-t)g_n \} \, \right\| \le t\left\| \, f-f_n \, \right\| +(1-t)\left\| \, g-g_n \, \right\| \rightarrow 0 \end{aligned}$$

as $n \rightarrow \infty$. Thus $tf+(1-t)g \in {\overline{W}}$, and hence ${\overline{W}}$ is convex. $\square$

We next show that $A: W \rightarrow W$.

### Lemma 3.2

Let $f \in W$. Then *Af* is continuous on *D*.

### Proof

Let $(T,\, x), \, (T_1,\, x_1) \in D$, and suppose $T<T_1<T_c$. We can deal with the case where $T_1=T_c$ similarly . Then

$$\begin{aligned} | Af(T,\, x)-Af(T_1,\, x_1) | \le | Af(T,\, x)-Af(T_1,\, x) |+| Af(T_1,\, x)-Af(T_1,\, x_1) |. \end{aligned}$$

*Step 1*. A straightforward calculation gives

$$\begin{aligned} Af(T,\, x)-Af(T_1,\, x)=\int _I U(x,\,\eta ) \, I_1 \, d\eta \, \int _I U(x,\,\xi ) \left\{ I_2+I_3+I_4 \right\} \, d\xi , \end{aligned}$$

where

$$\begin{aligned} I_1= & {} \sqrt{ \, \frac{f(T,\, \eta )}{\, \eta ^2+f(T,\, \eta )\,} \,} \tanh \frac{\,\sqrt{\,\eta ^2+f(T,\, \eta )\,}\,}{2T} + \sqrt{ \, \frac{f(T_1,\, \eta )}{\, \eta ^2+f(T_1,\, \eta )\,} \,} \tanh \frac{\,\sqrt{\,\eta ^2+f(T_1,\, \eta )\,}\,}{2T_1}, \\ I_2= & {} \frac{f(T,\, \xi )-f(T_1,\, \xi )}{ \, \sqrt{ f(T,\, \xi ) }+\sqrt{ f(T_1,\, \xi ) } \, } \frac{1}{\, \sqrt{ \xi ^2+f(T,\, \xi ) \,} \,} \tanh \frac{\,\sqrt{\,\xi ^2+f(T,\, \xi )\,}\,}{2T},\\ I_3= & {} \sqrt{ f(T_1,\, \xi ) } \left\{ \frac{1}{\, \sqrt{ \xi ^2+f(T,\, \xi ) \,} \,} \tanh \frac{\,\sqrt{\,\xi ^2+f(T,\, \xi )\,}\,}{2T} \right. \\&\left. \qquad \qquad \qquad \qquad -\frac{1}{\, \sqrt{ \xi ^2+f(T_1,\, \xi ) \,} \,} \tanh \frac{\,\sqrt{\,\xi ^2+f(T_1,\, \xi )\,}\,}{2T} \right\} ,\\ I_4= & {} \sqrt{ \, \frac{f(T_1,\, \xi )}{\, \xi ^2+f(T_1,\, \xi )\,} \,} \left\{ \tanh \frac{\,\sqrt{\, \xi ^2+f(T_1,\, \xi )\,}\,}{2T} - \tanh \frac{\,\sqrt{\, \xi ^2+f(T_1,\, \xi )\,}\,}{2T_1} \right\} . \end{aligned}$$

The function *f* is continuous since $f \in W$. Therefore, for an arbitrary $\varepsilon _1>0$, there is a $\delta >0$ such that if $|T_2-T_3|+|x_2-x_3|<\delta$, then $| f(T_2,\, x_2)-f(T_3,\, x_3) |<\varepsilon _1$. Here, $(T_2,\, x_2), (T_3,\, x_3) \in D$ are arbitrary and the $\delta >0$ does not depend on $(T_2,\, x_2), (T_3,\, x_3)$ since *f* is uniformly continuous on *D*. Since $f(T,\, \eta )/ f(T,\, \xi ) \le a$ by (2.2),

$$\begin{aligned} & \left| {\int_{I} U (x,{\mkern 1mu} \eta ){\mkern 1mu} I_{1} {\mkern 1mu} d\eta \times \int_{I} U (x,{\mkern 1mu} \xi ){\mkern 1mu} I_{2} {\mkern 1mu} d\xi } \right| \\ & \quad \le 2{\mkern 1mu} \frac{{{\mkern 1mu} U_{2}^{2} {\mkern 1mu} }}{{U_{1}^{2} }}\sqrt a \left\{ {\int_{I} {\frac{{U_{1} }}{{{\mkern 1mu} \sqrt {\eta ^{2} + \Delta _{1} (T)^{2} {\mkern 1mu} } {\mkern 1mu} }}} \tanh \frac{{{\mkern 1mu} \sqrt {{\mkern 1mu} \eta ^{2} + \Delta _{1} (T)^{2} {\mkern 1mu} } {\mkern 1mu} }}{{2T}}{\mkern 1mu} d\eta } \right\}^{2} \left| {f(T,{\mkern 1mu} \xi _{1} ) - f(T_{1} ,{\mkern 1mu} \xi _{1} )} \right| \\ & \quad \le 2{\mkern 1mu} \frac{{{\mkern 1mu} U_{2}^{2} {\mkern 1mu} }}{{U_{1}^{2} }}\sqrt a {\mkern 1mu} \varepsilon _{1} , \\ \end{aligned}$$

where $|T-T_1|<\delta$ with some $\xi _1 \in I$. Note that (see (1.3))

$$\begin{aligned} \int _I \frac{U_1}{\, \sqrt{ \eta ^2+\Delta _1(T)^2 \,} \,} \tanh \frac{\,\sqrt{\,\eta ^2+\Delta _1(T)^2 \,}\,}{2T} \,d\eta = 1, \quad T \in [0,\, \tau _1] \end{aligned}$$

and that

$$\begin{aligned} \int _I \frac{U_1}{\, \sqrt{ \eta ^2+0^2 \,} \,} \tanh \frac{\,\sqrt{\,\eta ^2+0^2 \,}\,}{2T} \,d\eta < 1, \quad T \in (\tau _1,\, T_c] \end{aligned}$$

with $$\Delta _1(T)=0$$ at $$T \in [\tau _1,\, T_c]$$. Since $$f(T,\, \xi )>f(T_1,\, \xi ) \;$$$$(T<T_1)$$,

$$\begin{aligned}&\left| \int _I U(x,\,\eta ) \, I_1 \, d\eta \times \int _I U(x,\,\xi ) \, I_3 \, d\xi \right| \\&\quad \le U_2 \, \Delta _2(0) \left\{ \int _I \frac{U_2}{\, \sqrt{ \eta ^2+\Delta _2(T)^2 \,} \,} \tanh \frac{\,\sqrt{\,\eta ^2+\Delta _2(T)^2 \,}\,}{2T} \,d\eta \right. \\&\qquad \left. +\int _I \frac{U_2}{\, \sqrt{ \eta ^2+\Delta _2(T_1)^2 \,} \,} \tanh \frac{\,\sqrt{\,\eta ^2+\Delta _2(T_1)^2 \,}\,}{2T_1} \,d\eta \right\} \\&\qquad \times \int _I \frac{1}{\, \xi ^2 \,} \,d\xi \, \left| f(T,\, \xi _1)-f(T_1,\, \xi _1) \right| \\&\quad \le 2 \, \frac{ \, U_2 \, \Delta _2(0)\,}{\varepsilon } \, \varepsilon _1, \end{aligned}$$

where $|T-T_1|<\delta$ with some $\xi _1 \in I$. Similarly,

$$\begin{gathered} \left| {\int_{I} U (x,{\mkern 1mu} \eta ){\mkern 1mu} I_{1} {\mkern 1mu} d\eta \times \int_{I} U (x,{\mkern 1mu} \xi ){\mkern 1mu} I_{4} {\mkern 1mu} d\xi } \right| \hfill \\ \qquad \le 2U_{2} {\mkern 1mu} \Delta _{2} (0){\mkern 1mu} \left( {\max _{{z \ge 0}} \frac{z}{{{\mkern 1mu} \cosh z{\mkern 1mu} }}} \right)^{2} \left\{ {\int_{I} {\frac{{U_{2} }}{{{\mkern 1mu} \sqrt {\eta ^{2} + \Delta _{2} (T)^{2} {\mkern 1mu} } {\mkern 1mu} }}} \tanh \frac{{{\mkern 1mu} \sqrt {{\mkern 1mu} \eta ^{2} + \Delta _{2} (T)^{2} {\mkern 1mu} } {\mkern 1mu} }}{{2T}}{\mkern 1mu} d\eta } \right. \hfill \\ \qquad \left. { + \int_{I} {\frac{{U_{2} }}{{{\mkern 1mu} \sqrt {\eta ^{2} + \Delta _{2} (T_{1} )^{2} {\mkern 1mu} } {\mkern 1mu} }}} \tanh \frac{{{\mkern 1mu} \sqrt {{\mkern 1mu} \eta ^{2} + \Delta _{2} (T_{1} )^{2} {\mkern 1mu} } {\mkern 1mu} }}{{2T_{1} }}{\mkern 1mu} d\eta } \right\} \hfill \\ \qquad \times \int_{I} {\frac{1}{{{\mkern 1mu} \xi {\mkern 1mu} }}} {\mkern 1mu} d\xi {\mkern 1mu} |T - T_{1} | \hfill \\ \qquad \le 4U_{2} {\mkern 1mu} \Delta _{2} (0){\mkern 1mu} \left( {\max _{{z \ge 0}} \frac{z}{{{\mkern 1mu} \cosh z{\mkern 1mu} }}} \right)^{2} {\mkern 1mu} (\ln \varepsilon ){\mkern 1mu} |T - T_{1} |\varepsilon _{1} , \hfill \\ \end{gathered}$$

where

$$\begin{aligned} |T-T_1|<\delta _1=\frac{\varepsilon _1}{\, 4 \, U_2 \, \Delta _2(0) \left( \max _{z \ge 0} \frac{z}{\, \cosh z \,} \right) ^2 \, \ln \varepsilon \,}. \end{aligned}$$

Thus

$$\begin{aligned} | Af(T,\, x)-Af(T_1,\, x) | \le \left( 2 \, \frac{ \, U_2^2 \,}{U_1^2} \sqrt{a}+ 2 \, \frac{ \, U_2 \, \Delta _2(0)\,}{\varepsilon }+1 \right) \, \varepsilon _1, \end{aligned}$$

where $$|T-T_1|<\min (\delta , \, \delta _1)$$.

*Step 2*. By hypothesis, the potential $U(\cdot ,\,\cdot )$ is continuous on the compact set $I^2$, and hence $U(\cdot ,\,\cdot )$ is uniformly continuous. Therefore, for an arbitrary $\varepsilon _1>0$, there is a $\delta _2>0$ such that if $|x-x_1|<\delta _2$, then $| U(x,\, \eta )-U(x_1,\, \eta ) |<\varepsilon _1$. Note that the $\delta _2$ does not depend on $f \in W$. A straightforward calculation gives

$$\begin{gathered} \left| {Af(T_{1} ,{\mkern 1mu} x) - Af(T_{1} ,{\mkern 1mu} x_{1} )} \right| \hfill \\ \qquad \le \int_{I} {\left\{ {U(x,{\mkern 1mu} \eta ) + U(x_{1} ,{\mkern 1mu} \eta )} \right\}} \sqrt {{\mkern 1mu} \frac{{f(T_{1} ,{\mkern 1mu} \eta )}}{{{\mkern 1mu} \eta ^{2} + f(T_{1} ,{\mkern 1mu} \eta ){\mkern 1mu} }}{\mkern 1mu} } \tanh \frac{{{\mkern 1mu} \sqrt {{\mkern 1mu} \eta ^{2} + f(T_{1} ,{\mkern 1mu} \eta ){\mkern 1mu} } {\mkern 1mu} }}{{2T_{1} }}{\mkern 1mu} d\eta \hfill \\ \qquad \times \int_{I} {\left| {U(x,{\mkern 1mu} \xi ) - U(x_{1} ,{\mkern 1mu} \xi )} \right|} \sqrt {{\mkern 1mu} \frac{{f(T_{1} ,{\mkern 1mu} \xi )}}{{{\mkern 1mu} \xi ^{2} + f(T_{1} ,{\mkern 1mu} \xi ){\mkern 1mu} }}{\mkern 1mu} } \tanh \frac{{{\mkern 1mu} \sqrt {{\mkern 1mu} \xi ^{2} + f(T_{1} ,{\mkern 1mu} \xi ){\mkern 1mu} } {\mkern 1mu} }}{{2T_{1} }}{\mkern 1mu} d\xi \hfill \\ \qquad \le 2\int_{I} {\mkern 1mu} U_{2} {\mkern 1mu} \sqrt {{\mkern 1mu} \frac{{\Delta _{2} (T_{1} )^{2} }}{{{\mkern 1mu} \eta ^{2} + \Delta _{2} (T_{1} )^{2} {\mkern 1mu} }}{\mkern 1mu} } \tanh \frac{{{\mkern 1mu} \sqrt {{\mkern 1mu} \eta ^{2} + \Delta _{2} (T_{1} )^{2} {\mkern 1mu} } {\mkern 1mu} }}{{2T_{1} }}{\mkern 1mu} d\eta \hfill \\ \qquad \times \int_{I} {\mkern 1mu} \left| {U(x,{\mkern 1mu} \xi ) - U(x_{1} ,{\mkern 1mu} \xi )} \right|{\mkern 1mu} \sqrt {{\mkern 1mu} \frac{{\Delta _{2} (T_{1} )^{2} }}{{{\mkern 1mu} \eta ^{2} + \Delta _{2} (T_{1} )^{2} {\mkern 1mu} }}{\mkern 1mu} } \tanh \frac{{{\mkern 1mu} \sqrt {{\mkern 1mu} \eta ^{2} + \Delta _{2} (T_{1} )^{2} {\mkern 1mu} } {\mkern 1mu} }}{{2T_{1} }}{\mkern 1mu} d\xi \hfill \\ \qquad \le 2\frac{{{\mkern 1mu} \Delta _{2} (0)^{2} {\mkern 1mu} }}{{U_{2} }}{\mkern 1mu} \varepsilon _{1} , \hfill \\ \end{gathered}$$

where $$|x-x_1|<\delta _2$$.

*Step 3*. Steps 1 and 2 thus imply

$$\begin{aligned} | Af(T,\, x)-Af(T_1,\, x_1) | \le \left( 2 \, \frac{ \, U_2^2 \,}{U_1^2} \sqrt{a}+ 2 \, \frac{ \, U_2 \, \Delta _2(0)\,}{\varepsilon }+1+ 2 \frac{\, \Delta _2(0)^2 \,}{U_2} \right) \, \varepsilon _1, \end{aligned}$$

where $|T-T_1|+|x-x_1|<\min (\delta , \, \delta _1, \delta _2)$. Therefore, *Af* is continuous on *D*. $\square$

### Lemma 3.3

Let $$f \in W$$.

1. *Af* is partially differentiable with respect to both *T* and *x*. Its first-order partial derivatives $(Af)_T$ and $(Af)_x$ are both continuous on *D*. Therefore, $Af \in C^1(D)$.
2. *Af* is twice partially differentiable with respect to both *T* and *x*. Its second-order partial derivatives $(Af)_{TT}$, $(Af)_{Tx}=(Af)_{xT}$ and $(Af)_{xx}$ are all continuous on *D*. Therefore, $Af \in C^2(D)$.

### Proof

(1) Let us show that *Af* is partially differentiable with respect to *T* at $(T_c,\,x_0) \in D$. Note that $Af(T_c,\,x_0)=0$. Let $T<T_c$. It follows from $f(T_c,\, \xi )=0$ (see (2.2)) that

$$\begin{aligned} f(T,\, \xi )=f(T_c,\, \xi )+(T-T_c)f_T(T_1,\, \xi )=(T_c-T)\left( -f_T(T_1,\, \xi ) \right) \end{aligned}$$

for some $T_1$ $(T<T_1<T_c)$. Then

$$\begin{aligned} \frac{\, Af(T_c,\,x_0)-Af(T,\,x_0) \,}{T_c-T}= & {} -\left( \int _I U(x_0,\,\xi ) \sqrt{ \, \frac{f(T,\, \xi )/(T_c-T)}{\, \xi ^2+f(T,\, \xi )\,} \,} \, \tanh \frac{\,\sqrt{\,\xi ^2+f(T,\, \xi )\,}\,}{2T}\, d\xi \right) ^2 \\= & {} -\left( \int _I U(x_0,\,\xi ) \sqrt{ \, \frac{-f_T(T_1,\, \xi )}{\, \xi ^2+f(T,\, \xi )\,} \,} \, \tanh \frac{\,\sqrt{\,\xi ^2+f(T,\, \xi )\,}\,}{2T}\, d\xi \right) ^2. \end{aligned}$$

 Since *T* is in a neighborhood of $T_c$, we let $T \ge T_c/2$. Therefore,

$$\begin{aligned} \sqrt{ \, \frac{-f_T(T_1,\, \xi )}{\, \xi ^2+f(T,\, \xi )\,} \,} \, \tanh \frac{\,\sqrt{\,\xi ^2+f(T,\, \xi )\,}\,}{2T} \le \frac{ \, \sqrt{ M_T} \, }{\xi } \, \tanh \frac{\, \xi \,}{T_c}, \end{aligned}$$

where the right side is independent of *T* and is Lebesgue integrable on *I*. Thus, as $T \uparrow T_c$,

$$\begin{aligned} \frac{\, Af(T_c,\,x_0)-Af(T,\,x_0) \,}{T_c-T} \rightarrow -\left( \int _I U(x_0,\,\xi ) \frac{ \, \sqrt{ -f_T(T_c,\, \xi ) } \, }{\xi } \, \tanh \frac{\, \xi \,}{\, 2T_c \,} \, d\xi \right) ^2. \end{aligned}$$

 Therefore, *Af* is partially differentiable with respect to *T* at $(T_c,\,x_0)$, and

3.1 $$\begin{aligned} (Af)_T(T_c,\,x_0)=-\left( \int _I U(x_0,\,\xi ) \frac{ \, \sqrt{ -f_T(T_c,\, \xi ) } \, }{\xi } \, \tanh \frac{\, \xi \,}{\, 2T_c \,} \, d\xi \right) ^2. \end{aligned}$$

 We next show that $(Af)_T$ is continuous at $(T_c,\,x_0)$. Here,

3.2 $$\begin{aligned} (Af)_T(T,\,x)= & {} \int _I U(x,\,\eta ) \sqrt{ \, \frac{f(T,\, \eta )}{\, \eta ^2+f(T,\, \eta )\,} \,} \, \tanh \frac{\,\sqrt{\,\eta ^2+f(T,\, \eta )\,}\,}{2T}\, d\eta \\ \nonumber&\quad \times \int _I U(x,\,\xi ) \left( J_1+J_2+J_3 \right) \, d\xi , \end{aligned}$$

where

$$\begin{aligned} J_1= & {} \frac{\, f_T(T,\,\xi ) \,}{\, \sqrt{ f(T,\,\xi )} \,} \, \frac{\xi ^2}{\, \left\{ \, \xi ^2+f(T,\, \xi ) \, \right\} ^{3/2}\, } \tanh \frac{ \, \sqrt{ \, \xi ^2+f(T,\, \xi ) \, } \,}{2T}, \\ J_2= & {} \frac{\sqrt{ f(T,\, \xi ) } \, f_T(T,\, \xi )}{\, 2T \left\{ \, \xi ^2+f(T,\, \xi ) \, \right\} \cosh ^2 \frac{ \, \sqrt{ \, \xi ^2+f(T,\, \xi ) \, } \, }{2T} \,}, \\ J_3= & {} -\frac{ \sqrt{ f(T,\, \xi ) } }{\, T^2 \cosh ^2 \frac{ \, \sqrt{ \, \xi ^2+f(T,\, \xi ) \, } \, }{2T} \,}. \end{aligned}$$

Note that

3.3 $$\begin{aligned} (Af)_T(T,\,x)-(Af)_T(T_c,\,x_0)= & {} (Af)_T(T,\,x)-(Af)_T(T_c,\,x) \nonumber \\&+ (Af)_T(T_c,\,x)-(Af)_T(T_c,\,x_0). \end{aligned}$$

In order to show that $(Af)_T(T,\,x) \rightarrow (Af)_T(T_c,\,x)$ as $T \uparrow T_c$, we show that as $T \uparrow T_c$,

$$\begin{gathered} \int_{I} U (x,{\mkern 1mu} \eta )\sqrt {{\mkern 1mu} \frac{{f(T,{\mkern 1mu} \eta )}}{{{\mkern 1mu} \eta ^{2} + f(T,{\mkern 1mu} \eta ){\mkern 1mu} }}{\mkern 1mu} } {\mkern 1mu} \tanh \frac{{{\mkern 1mu} \sqrt {{\mkern 1mu} \eta ^{2} + f(T,{\mkern 1mu} \eta ){\mkern 1mu} } {\mkern 1mu} }}{{2T}}{\mkern 1mu} d\eta \int_{I} U (x,{\mkern 1mu} \xi ){\mkern 1mu} J_{1} {\mkern 1mu} d\xi \to (Af)_{T} (T_{c} ,{\mkern 1mu} x), \hfill \\ \int_{I} U (x,{\mkern 1mu} \eta )\sqrt {{\mkern 1mu} \frac{{f(T,{\mkern 1mu} \eta )}}{{{\mkern 1mu} \eta ^{2} + f(T,{\mkern 1mu} \eta ){\mkern 1mu} }}{\mkern 1mu} } {\mkern 1mu} \tanh \frac{{{\mkern 1mu} \sqrt {{\mkern 1mu} \eta ^{2} + f(T,{\mkern 1mu} \eta ){\mkern 1mu} } {\mkern 1mu} }}{{2T}}{\mkern 1mu} d\eta \int_{I} U (x,{\mkern 1mu} \xi ){\mkern 1mu} (J_{2} + J_{3} ){\mkern 1mu} d\xi \to 0. \hfill \\ \end{gathered}$$

A straightforward calculation gives

$$\begin{aligned} U(x,\,\eta ) \sqrt{ \, \frac{f(T,\, \eta )}{\, \eta ^2+f(T,\, \eta )\,} \,} \, \tanh \frac{\,\sqrt{\,\eta ^2+f(T,\, \eta )\,}\,}{2T} \; U(x,\,\xi ) \, J_1 \le U_2^2 \, \sqrt{a} \left( \frac{1}{\, \eta \,} \tanh \frac{\, \eta \,}{T_c} \right) ^2 M_T. \end{aligned}$$

Here, we assumed $T \ge T_c/2$. The right side of this inequality is independent of *T* and is Lebesgue integrable on $I^2$, and so (as $T \uparrow T_c$)

$$\begin{aligned} \int _I U(x,\,\eta ) \sqrt{ \, \frac{f(T,\, \eta )}{\, \eta ^2+f(T,\, \eta )\,} \,} \, \tanh \frac{\,\sqrt{\,\eta ^2+f(T,\, \eta )\,}\,}{2T}\, d\eta \int _I U(x,\,\xi ) \, J_1 \, d\xi \rightarrow (Af)_T(T_c,\,x). \end{aligned}$$

 Similarly we can show that

$$\begin{aligned} \int _I U(x,\,\eta ) \sqrt{ \, \frac{f(T,\, \eta )}{\, \eta ^2+f(T,\, \eta )\,} \,} \, \tanh \frac{\,\sqrt{\,\eta ^2+f(T,\, \eta )\,}\,}{2T}\, d\eta \int _I U(x,\,\xi ) \, (J_2+J_3) \, d\xi \rightarrow 0 \end{aligned}$$

as $T \uparrow T_c$. Moreover, we have similarly that

$$\begin{aligned} (Af)_T(T_c,\,x) \rightarrow (Af)_T(T_c,\,x_0) \end{aligned}$$

as $x \rightarrow x_0$. It thus follows from (3.3) that $(Af)_T$ is continuous at $(T_c,\,x_0)$. Similarly we can show the rest of (1), and (2). Note that $(Af)_{TT}$ is given as follows.

$$\begin{aligned} \quad (Af)_{TT}(T,\,x)= & {} \frac{1}{\, 2 \,} \left\{ \int _I U(x,\,\eta ) \, \left( J_1+J_2+J_3 \right) \, d\eta \right\} ^2 \\&+\int _I U(x,\,\eta ) \sqrt{ \, \frac{f(T,\, \eta )}{\, \eta ^2+f(T,\, \eta )\,} \,} \, \tanh \frac{\,\sqrt{\,\eta ^2+f(T,\, \eta )\,}\,}{2T}\, d\eta \\&\times \int _I U(x,\,\xi ) \left\{ K_1+ \frac{1}{\, \cosh ^2 \frac{ \, \sqrt{ \, \xi ^2+f(T,\, \xi ) \, } \, }{2T} \,} \left( K_2+K_3+K_4+K_5 \right) \right\} \, d\xi , \end{aligned}$$

where

$$\begin{aligned} K_1= & {} \left\{ \frac{\, f_{TT}(T,\,\xi ) \,}{\, \sqrt{ f(T,\,\xi )} \,} -\frac{\, f_T(T,\,\xi )^2 \,}{\, 2\sqrt{ f(T,\,\xi )}^3 \,} -\frac{\, 3 f_T(T,\,\xi )^2 \,}{\, 2\sqrt{ f(T,\,\xi )} \left( \, \xi ^2+f(T,\, \xi ) \, \right) \,} \right\} \\&\qquad \times \frac{\xi ^2}{\, \left\{ \, \xi ^2+f(T,\, \xi ) \, \right\} ^{3/2}\, } \tanh \frac{ \, \sqrt{ \, \xi ^2+f(T,\, \xi ) \, } \,}{2T}, \\ K_2= & {} 
\frac{\, f_T(T,\,\xi ) \,}{\, 2\sqrt{ f(T,\,\xi )} \,} \frac{\xi ^2}{\, \xi ^2+f(T,\, \xi ) \, } \left\{ \frac{\, f_T(T,\,\xi ) \,}{\, 2T \, ( \xi ^2+f(T,\, \xi ) ) \,} -\frac{1}{\, T^2 \,} \right\} , \\ K_3= & {} \frac{\, f_T(T,\,\xi ) \,}{\, 2\sqrt{ f(T,\,\xi )} \,} \left\{ \frac{\, f_T(T,\,\xi ) \,}{\, 2T \, ( \xi ^2+f(T,\, \xi ) ) \,} -\frac{1}{\, T^2 \,} \right\} , \\ K_4= & {} \sqrt{ f(T,\,\xi ) \,} \left\{ \frac{\, f_{TT}(T,\,\xi ) \,}{\, 2T \, ( \xi ^2+f(T,\, \xi ) ) \,} -\frac{\, f_T(T,\,\xi ) \,}{\, 2T^2 \, ( \xi ^2+f(T,\, \xi ) ) \,} -\frac{\, (f_T(T,\,\xi ))^2 \,}{\, 2T \, ( \xi ^2+f(T,\, \xi ) )^2 \,} +\frac{2}{\, T^3 \,} \right\} , \\ K_5= & {} -\sqrt{ f(T,\,\xi ) \,} \, \sqrt{ \, \xi ^2+f(T,\, \xi ) \, } \left\{ \frac{\, f_T(T,\,\xi ) \,}{\, 2T \, ( \xi ^2+f(T,\, \xi ) ) \,} -\frac{1}{\, T^2 \,} \right\} ^2 \tanh \frac{ \, \sqrt{ \, \xi ^2+f(T,\, \xi ) \, } \,}{2T}. \end{aligned}$$

$$\square$$

A proof similar to that of^20^[Lemma 3.4] gives the following.

### Lemma 3.4

Let $f \in W$. Then $\Delta _1(T)^2 \le Af(T,\,x) \le \Delta _2(T)^2$ at each $(T,\,x) \in D$.

### Lemma 3.5

Let $f \in W$. Then $Af(T_c,\, x)=0$ $(x \in I)$, and

$$\begin{aligned} \frac{Af(T,\,x)}{\,Af(T,\,x_1) \,} \le a. \end{aligned}$$

### Proof

By (2.2),

$$\begin{aligned} Af(T_c,\,x)=\left( \int _I U(x,\,\xi ) \sqrt{ \, \frac{f(T_c,\, \xi )}{\, \xi ^2+f(T_c,\, \xi )\,} \,} \, \tanh \frac{\,\sqrt{\,\xi ^2+f(T_c,\, \xi )\,}\,}{2T_c}\, d\xi \right) ^2=0. \end{aligned}$$

Next, it follows from (1.2) that at $T \in [0,\, T_c)$,

$$\begin{aligned} \frac{Af(T,\,x)}{\,Af(T,\,x_1) \,} = \left( \frac{U(x,\, \xi _1)}{\, U(x_1,\, \xi _2) \,} \right) ^2 \le \left( \frac{ \displaystyle { \max _{(x,\,\xi ) \in I^2} U(x,\,\xi ) }}{\, \displaystyle { \min _{(x,\,\xi ) \in I^2} U(x,\,\xi ) } \,} \right) ^2 = a, \end{aligned}$$

where $\xi _1, \, \xi _2 \in I$. It then follows from Remark 2.2 and (3.1) that at $T=T_c$,

$$\begin{aligned} \frac{Af(T_c,\, x)}{\,Af(T_c,\, x_1) \,}= \frac{(Af)_T(T_c,\, x)}{\, (Af)_T(T_c,\, x_1) \,} \le \left( \frac{ \displaystyle { \max _{(x,\,\xi ) \in I^2} U(x,\,\xi ) }}{\, \displaystyle { \min _{(x,\,\xi ) \in I^2} U(x,\,\xi ) } \,} \right) ^2 =a. \end{aligned}$$

The result follows. $\square$

### Lemma 3.6

For $$f \in W$$, $$\displaystyle { -(Af)_T(T,\,x)>0. }$$ 

### Proof

It follows immediately from (3.2) that $-(Af)_T(T,\,x)>0$.$\square$

### Lemma 3.7

For $$f \in W$$,

$$\begin{aligned} \sup _{f \in W} \left[ \max _{(T,\, x) \in D} \left\{ -(Af)_T(T,\,x) \right\} \right] \le \sup _{f \in W} \left[ \max _{(T,\, x) \in D} \left\{ -f_T(T,\,x) \right\} \right] \; \big (=M_T \big ). \end{aligned}$$

### Proof

From (3.2) it follows that

$$\begin{aligned} -(Af)_T(T,\,x) \le \sqrt{a}\, A \left\{ B\sup _{f \in W} \left[ \max _{(T,\, x) \in D} \left\{ -f_T(T,\,x) \right\} \right] +C \right\} , \end{aligned}$$

where

$$\begin{aligned} A= & {} \int _I \frac{ \, U(x,\,\eta ) \,}{\, \sqrt{ \, \eta ^2+f(T,\, \eta )\,} \,} \, \tanh \frac{\,\sqrt{\,\eta ^2+f(T,\, \eta )\,}\,}{2T}\, d\eta , \\ B= & {} \int _I \frac{ \, U(x,\,\xi ) \,}{\, \sqrt{ \, \xi ^2+f(T,\, \xi )\,} \,} \left\{ \frac{\xi ^2}{\, \xi ^2+f(T,\, \xi ) \, }\, \tanh \frac{\,\sqrt{\,\xi ^2+f(T,\, \xi )\,}\,}{2T}\right. \\&\qquad \left. +\frac{f(T,\, \xi )}{\, \xi ^2+f(T,\, \xi ) \, }\, \frac{\sqrt{ \, \xi ^2+f(T,\, \xi )\,}}{\, 2T \,\cosh ^2 \frac{\,\sqrt{\,\xi ^2+f(T,\, \xi )\,}\,}{2T} \,} \right\} \, d\xi , \\ C= & {} \int _I U(x,\,\xi ) \, \frac{ \, f(T,\, \xi ) \,}{\, T^2 \, \cosh ^2 \frac{\,\sqrt{\,\xi ^2+f(T,\, \xi )\,}\,}{2T}\, }\, d\xi . \end{aligned}$$

Note that the function $\displaystyle { z \mapsto \frac{\,\tanh z \,}{z} }$ is strictly decreasing at $z>0$. It then follows from (2.3) that

3.4 $$\begin{aligned} a^{1/4}A \le a^{1/4} \max _{(T,\, x) \in D} \left[ \, \int _I \frac{\, U(x,\, \eta ) \,}{\eta } \, \tanh \frac{\eta }{\, 2T\,}\,d\eta \, \right] \le 1. \end{aligned}$$

First let $T<T_c$. Then $B<A$ since $\displaystyle { \tanh z>\frac{z}{\cosh z} }$ at $z>0$, $f(T,\, \xi )>0$ and $\xi \ge \varepsilon$. Therefore, $\sqrt{a}\, AB<( a^{1/4}A)^2\le 1$ by (3.4). Thus

$$\begin{aligned} -(Af)_T(T,\,x) \le \sqrt{a}\, A \left\{ B\sup _{f \in W} \left[ \max _{(T,\, x) \in D} \left\{ -f_T(T,\,x) \right\} \right] +C \right\} \le \sup _{f \in W} \left[ \max _{(T,\, x) \in D} \left\{ -f_T(T,\,x) \right\} \right] \end{aligned}$$

as long as

$$\begin{aligned} \sup _{f \in W} \left[ \max _{(T,\, x) \in D} \left\{ -f_T(T,\,x) \right\} \right] \ge \frac{\sqrt{a} \, AC}{\, 1-\sqrt{a}\, AB \,}. \end{aligned}$$

This inequality holds true since (see the definition of $M_T$ in (2.2))

$$\begin{aligned} \frac{\sqrt{a} \, AC}{\, 1-\sqrt{a}\, AB \,} = \frac{\sqrt{a} \, AC}{\, 1-\sqrt{a}\, A(A-B') \,} \le \frac{\sqrt{a} \, AC}{\, \sqrt{a}\, AB' \,}=\frac{C}{\,B' \,} \le M_T = \sup _{f \in W} \left[ \max _{(T,\, x) \in D} \left\{ -f_T(T,\,x) \right\} \right] . \end{aligned}$$

Here,

$$\begin{aligned} B'=\int _I \frac{ \, U(x,\,\xi ) \,}{\, \sqrt{ \, \xi ^2+f(T,\, \xi )\,} \,} \frac{f(T,\, \xi )}{\, \xi ^2+f(T,\, \xi ) \, }\left\{ \tanh \frac{\,\sqrt{\,\xi ^2+f(T,\, \xi )\,}\,}{2T} -\frac{\sqrt{ \, \xi ^2+f(T,\, \xi )\,}}{\, 2T \,\cosh ^2 \frac{\,\sqrt{\,\xi ^2+f(T,\, \xi )\,}\,}{2T} \,} \right\} \, d\xi . \end{aligned}$$

Note also that $1-\sqrt{a}\, A^2 \ge 0$. Thus, at $T<T_c$,

$$\begin{aligned} -(Af)_T(T,\,x) \le \sup _{f \in W} \left[ \max _{(T,\, x) \in D} \left\{ -f_T(T,\,x) \right\} \right] . \end{aligned}$$

Next let $T=T_c$. Then

$$\begin{aligned} - (Af)_{T} (T_{c} ,{\mkern 1mu} x) = & \left( {\int_{I} U (x,{\mkern 1mu} \xi )\frac{{{\mkern 1mu} \sqrt { - f_{T} (T_{c} ,{\mkern 1mu} \xi )} {\mkern 1mu} }}{{{\mkern 1mu} \xi {\mkern 1mu} }}{\mkern 1mu} \tanh \frac{{{\mkern 1mu} \xi {\mkern 1mu} }}{{2T_{c} }}{\mkern 1mu} d\xi } \right)^{2} \\ & \le \sqrt a \left( {\int_{I} U (x,{\mkern 1mu} \xi )\frac{{{\mkern 1mu} \sqrt { - f_{T} (T_{c} ,{\mkern 1mu} \xi )} {\mkern 1mu} }}{{{\mkern 1mu} \xi {\mkern 1mu} }}{\mkern 1mu} \tanh \frac{{{\mkern 1mu} \xi {\mkern 1mu} }}{{2T_{c} }}{\mkern 1mu} d\xi } \right)^{2} \\ & \le \sqrt a \left( {\int_{I} U (x,{\mkern 1mu} \xi )\frac{{{\mkern 1mu} 1{\mkern 1mu} }}{{{\mkern 1mu} \xi {\mkern 1mu} }}{\mkern 1mu} \tanh \frac{{{\mkern 1mu} \xi {\mkern 1mu} }}{{2T_{c} }}{\mkern 1mu} d\xi } \right)^{2} \mathop {\sup }\limits_{{f \in W}} \left[ {\max _{{(T,{\kern 1pt} x) \in D}} \left\{ { - f_{T} (T,{\mkern 1mu} x)} \right\}} \right] \\& \le \mathop {\sup }\limits_{{f \in W}} \left[ {\max _{{(T,{\kern 1pt} x) \in D}} \left\{ { - f_{T} (T,{\mkern 1mu} x)} \right\}} \right]. \\ \end{aligned}$$

 This is because at $T=T_c$,

$$\begin{aligned} \sqrt{a}\, A^2=\left( a^{1/4}\, \int _I \frac{\, U(x,\,\eta ) \,}{\, \eta \,} \, \tanh \frac{\, \eta \,}{2T_c}\, d\eta \right) ^2 \le 1 \end{aligned}$$

by (3.4). Thus

$$\begin{aligned} \sup _{f \in W} \left[ \max _{(T,\, x) \in D} \left\{ -(Af)_T(T,\,x) \right\} \right] \le \sup _{f \in W} \left[ \max _{(T,\, x) \in D} \left\{ -f_T(T,\,x) \right\} \right] =M_T. \end{aligned}$$

$$\square$$

### Remark 3.8

Let $U(x,\,\xi )=U_1=U_2$ at all $(x,\, \xi ) \in I^2$. Then, $a=1$ and $f(T,\, x)=\Delta _1(T)^2=\Delta _2(T)^2$. Moreover, $T_c=\tau _1=\tau _2$ and

$$\begin{aligned} a^{1/4}A=\int _I \frac{U_2}{\,\sqrt{\,\xi ^2+\Delta _2(T)^2\,}\,} \, \tanh \frac{\, \sqrt{\,\xi ^2+\Delta _2(T)^2\,}\,}{2T}\,d\xi =1 \end{aligned}$$

at all $(T,\, x) \in [0,\, T_c] \times I$ (see (1.5)). Therefore, the preceding lemma holds true not only when the potential $U(\cdot ,\,\cdot )$ in the BCS-Bogoliubov gap equation (1.1) is a positive constant, but also when $U(\cdot ,\,\cdot )$ is a function.

### Lemma 3.9

The set *AW* is equicontinuous.

### Proof

Let $f \in W$. Let $(T,\, x), \, (T_1,\, x_1) \in D$ and suppose $T<T_1<T_c$. We can deal with the case where $T_1=T_c$ similarly. Then

$$\begin{aligned} | Af(T,\, x)-Af(T_1,\, x_1) | \le | Af(T,\, x)-Af(T_1,\, x) |+| Af(T_1,\, x)-Af(T_1,\, x_1) |. \end{aligned}$$

 The preceding lemma gives

$$\begin{aligned} | f(T,\, \xi )-f(T_1,\, \xi ) | = \left| f_T(T_2, \, \xi ) \right| \cdot \left| T-T_1 \right| \le M_T | T-T_1 |. \end{aligned}$$

Here, $T<T_2<T_1$ and $\xi \in I$. Therefore, a proof similar to that of Lemm 3.2 gives

$$\begin{aligned} | Af(T,\, x)-Af(T_1,\, x_1) |\le & {} \left\{ \left( 2 \, \frac{ \, U_2^2 \,}{U_1^2} \sqrt{a}+ 2 \, \frac{ \, U_2 \, \Delta _2(0)\,}{\varepsilon } \right) M_T \right. \\&+ \; 4 \, U_2 \, \Delta _2(0) \left( \max _{z \ge 0} \frac{z}{\, \cosh z \,} \right) ^2 \, \ln \varepsilon \\&\left. +2 \; \frac{\, \Delta _2(0)^2 \,}{U_2}\, \max _{(x,\, \xi ) \in I^2} \left\{ U_x(x,\,\xi ) \right\} \right\} \, \left( | T-T_1 | +| x-x_1 | \right) , \end{aligned}$$

from which the result follows. $\square$

Since $Af(T,\, x)\le \Delta _2(T)^2 \le \Delta _2(0)^2$ for $f \in W$ (see Lemma 3.4), the set *AW* is uniformly bounded. Moreover, *AW* is equicontinuous by the preceding lemma. We thus have the following.

### Lemma 3.10

$\displaystyle { A: \, W \rightarrow W}$, and the set *AW* is relatively compact.

### Lemma 3.11

The operator $A:\, W \rightarrow W$ is continuous.

### Proof

Let $T<T_c$. Then, for $f, \, g \in W$,

$$\begin{aligned} Af(T,\, x)-Ag(T,\, x)=\int _I U(x,\,\eta ) \, L_1 \, d\eta \, \int _I U(x,\,\xi ) \left\{ L_2+L_3 \right\} \, d\xi , \end{aligned}$$

where

$$\begin{aligned} L_1= & {} \sqrt{ \, \frac{f(T,\, \eta )}{\, \eta ^2+f(T,\, \eta )\,} \,} \tanh \frac{\,\sqrt{\,\eta ^2+f(T,\, \eta )\,}\,}{2T} + \sqrt{ \, \frac{g(T,\, \eta )}{\, \eta ^2+g(T,\, \eta )\,} \,} \tanh \frac{\,\sqrt{\,\eta ^2+g(T,\, \eta )\,}\,}{2T}, \\ L_2= & {} \frac{f(T,\, \xi )-g(T,\, \xi )}{ \, \sqrt{ f(T,\, \xi ) }+\sqrt{ g(T,\, \xi ) } \, } \frac{1}{\, \sqrt{ \xi ^2+f(T,\, \xi ) \,} \,} \tanh \frac{\,\sqrt{\,\xi ^2+f(T,\, \xi )\,}\,}{2T},\\ L_3= & {} \sqrt{ g(T,\, \xi ) } \left\{ \frac{1}{\, \sqrt{ \xi ^2+f(T,\, \xi ) \,} \,} \tanh \frac{\,\sqrt{\,\xi ^2+f(T,\, \xi )\,}\,}{2T} \right. \\&\left. \qquad \qquad \qquad \qquad -\frac{1}{\, \sqrt{ \xi ^2+g(T,\, \xi ) \,} \,} \tanh \frac{\,\sqrt{\,\xi ^2+g(T,\, \xi )\,}\,}{2T} \right\} . \end{aligned}$$

 Since $f(T,\, \eta )/ f(T,\, \xi ) \le a$ and $g(T,\, \eta )/ g(T,\, \xi )\le a$ by (2.2), it follows

$$\begin{aligned} &\left| {\int_{I} U (x,{\mkern 1mu} \eta ){\mkern 1mu} L_{1} {\mkern 1mu} d\eta \times \int_{I} U (x,{\mkern 1mu} \xi ){\mkern 1mu} L_{2} {\mkern 1mu} d\xi } \right| \\ & \quad \le 2{\mkern 1mu} \frac{{{\mkern 1mu} U_{2}^{2} {\mkern 1mu} }}{{U_{1}^{2} }}\sqrt a \left\{ {\int_{I} {\frac{{U_{1} }}{{{\mkern 1mu} \sqrt {\eta ^{2} + \Delta _{1} (T)^{2} {\mkern 1mu} } {\mkern 1mu} }}} \tanh \frac{{{\mkern 1mu} \sqrt {{\mkern 1mu} \eta ^{2} + \Delta _{1} (T)^{2} {\mkern 1mu} } {\mkern 1mu} }}{{2T}}{\mkern 1mu} d\eta } \right\}^{2} \left\|f - g\right\| \\ & \quad \le 2{\mkern 1mu} \frac{{{\mkern 1mu} U_{2}^{2} {\mkern 1mu} }}{{U_{1}^{2} }}\sqrt a {\mkern 1mu} \left\|f - g\right\|. \\ \end{aligned}$$

 Moreover,

$$\begin{aligned} & \left| {\int_{I} U (x,\eta )L_{1} d\eta \times \int_{I} U (x,\xi )L_{3} d\xi } \right| \\ & \quad \le 2\frac{{U_{2}^{2} \Delta _{2} (0)^{2} }}{{U_{1} }}\int_{I} {\frac{{U_{1} }}{{\sqrt {\eta ^{2} + \Delta _{1} (T)^{2} } }}} \tanh \frac{{\sqrt {\eta ^{2} + \Delta _{1} (T)^{2} } }}{{2T}}d\eta \int_{I} {\frac{1}{{\xi ^{3} }}} d\xi \left\| {f - g} \right\| \\ & \quad \le \frac{{U_{2}^{2} \Delta _{2} (0)^{2} }}{{U_{1} \varepsilon ^{2} }}\left\| {f - g} \right\|. \\ \end{aligned}$$

 Therefore, at $$T<T_c$$,

$$\left| {Af(T,{\mkern 1mu} x) - Ag(T,{\mkern 1mu} x)} \right| \le \left( {2{\mkern 1mu} \frac{{{\mkern 1mu} U_{2}^{2} {\mkern 1mu} }}{{U_{1}^{2} }}\sqrt a + \frac{{{\mkern 1mu} U_{2}^{2} {\mkern 1mu} \Delta _{2} (0)^{2} {\mkern 1mu} }}{{U_{1} {\mkern 1mu} \varepsilon ^{2} }}} \right){\mkern 1mu} \left\| {f - g} \right\|.$$

 Since $Af(T_c,\, x)=Ag(T_c,\, x)=0$, this inequality holds true also at $T=T_c$. Thus

$$\begin{aligned} \Vert Af-Ag \Vert \le \left( 2 \, \frac{ \, U_2^2 \,}{U_1^2} \sqrt{a}+ \frac{ \, U_2^2 \, \Delta _2(0)^2 \,}{U_1 \, \varepsilon ^2} \right) \, \Vert f-g \Vert . \end{aligned}$$

The result follows. $\square$

We next extend the domain *W* of our operator *A* to its closure ${\overline{W}}$ with respect to the norm $\Vert \cdot \Vert$ of the Banach space *C*(*D*).

### Lemma 3.12

 $$\displaystyle { A: \, {\overline{W}} \rightarrow {\overline{W}}}$$.

### Proof

For $f \in {\overline{W}}$, there is a sequence $\{ f_n \}_{n=1}^{\infty } \subset W$ satisfying $\Vert f-f_n \Vert \rightarrow 0$ as $n \rightarrow \infty$. By the preceding lemma,

$$\begin{aligned} \Vert Af_n-Af_m \Vert \le \left( 2 \, \frac{ \, U_2^2 \,}{U_1^2} \sqrt{a}+ \frac{ \, U_2^2 \, \Delta _2(0)^2 \,}{U_1 \, \varepsilon ^2} \right) \, \Vert f_n-f_m \Vert . \end{aligned}$$

 Therefore, the sequence $\{ Af_n \}_{n=1}^{\infty } \subset W$ is a Cauchy sequence. Hence there is an element $F \in {\overline{W}}$ satisfying $\Vert F-Af_n \Vert \rightarrow 0$ as $n \rightarrow \infty$. Note that the element *F* does not depend on how to choose the sequence $\{ f_n \}_{n=1}^{\infty } \subset W$, as shown below. Suppose that there is another sequence $\{ g_n \}_{n=1}^{\infty } \subset W$ satisfying $\Vert f-g_n \Vert \rightarrow 0$ as $n \rightarrow \infty$. Similarly, the sequence $\{ Ag_n \}_{n=1}^{\infty } \subset W$ becomes a Cauchy sequence, and hence there is an element $G \in {\overline{W}}$ satisfying $\Vert G-Ag_n \Vert \rightarrow 0$ as $n \rightarrow \infty$. Then

$$\begin{aligned} \Vert F-G \Vert \le \Vert F-Af_n \Vert +\Vert Af_n-Ag_n \Vert +\Vert Ag_n-G \Vert \rightarrow 0 \end{aligned}$$

as $n \rightarrow \infty$. Therefore, $F=G$, and hence *F* does not depend on how to choose the sequence in *W*. Thus we define $F=Af$. The result thus follows. $\square$

### Lemma 3.13

For $$f \in {\overline{W}}$$,

$$\begin{aligned} Af(T,\,x)=\left( \int _I U(x,\,\xi ) \sqrt{ \, \frac{f(T,\, \xi )}{\, \xi ^2+f(T,\, \xi )\,} \,} \, \tanh \frac{\,\sqrt{\,\xi ^2+f(T,\, \xi )\,}\,}{2T}\, d\xi \right) ^2. \end{aligned}$$

### Proof

For $f \in {\overline{W}}$, there is a sequence $\{ f_n \}_{n=1}^{\infty } \subset W$ satisfying $\Vert f-f_n \Vert \rightarrow 0$ as $n \rightarrow \infty$. Since *f* is Lebesgue integrable on *I*, we set

$$\begin{aligned} H(T,\, x)=\left( \int _I U(x,\,\xi ) \sqrt{ \, \frac{f(T,\, \xi )}{\, \xi ^2+f(T,\, \xi )\,} \,} \, \tanh \frac{\,\sqrt{\,\xi ^2+f(T,\, \xi )\,}\,}{2T}\, d\xi \right) ^2 \end{aligned}$$

at all $(T,\, x) \in D$. Then

$$\begin{aligned} \left| Af(T,\,x)-H(T,\, x) \right|\le & {} \left| Af(T,\,x)-Af_n(T,\, x) \right| +\left| Af_n(T,\,x)-H(T,\, x) \right| \\\le & {} \left\| Af-Af_n \right\| +\left| Af_n(T,\,x)-H(T,\, x) \right| . \end{aligned}$$

 By the proof of Lemma 3.12,

$$\begin{aligned} \left\| Af-Af_n \right\| \rightarrow 0 \end{aligned}$$

as $n \rightarrow \infty$. On the other hand, a proof similar to that of Lemma 3.11 gives

$$\begin{aligned} \left| Af_n(T,\,x)-H(T,\, x) \right| \le \left( 2 \, \frac{ \, U_2^2 \,}{U_1^2} \sqrt{a}+ \frac{ \, U_2^2 \, \Delta _2(0)^2 \,}{U_1 \, \varepsilon ^2} \right) \, \Vert f_n-f \Vert \rightarrow 0 \end{aligned}$$

as $n \rightarrow \infty$. The result thus follows. $\square$

A straightforward calculation gives the following.

### Lemma 3.14

 $\displaystyle { A: \, {\overline{W}} \rightarrow {\overline{W}}}$ is continuous. Moreover, the set $A{\overline{W}}$ is uniformly bounded and equicontinuous, and hence the set $A{\overline{W}}$ is relatively compact.

Lemma 3.14 immediately implies the following.

### Lemma 3.15

The operator $A:\, {\overline{W}} \rightarrow {\overline{W}}$ is compact. Therefore, the operator $A:\, {\overline{W}} \rightarrow {\overline{W}}$ has a unique fixed point $f_0 \in {\overline{W}}$, i.e., $\displaystyle { f_0=Af_0 }$.

### Proof

Applying the Schauder fixed-point theorem gives that the operator $A:\, {\overline{W}} \rightarrow {\overline{W}}$ has at least one fixed point $f_0 \in {\overline{W}}$. A proof similar to that of^20^[Lemma 3.10] gives the uniqueness of $f_0 \in {\overline{W}}$. $\square$

Our proof of Theorem 2.5 is now complete.

In order to give a proof of Theorem 2.8, we need to deal with the thermodynamic potential $\Omega$ and differentiate it with respect to the temperature *T* twice, as mentioned before. Note that the thermodynamic potential $\Omega$ has the fixed point $f_0 \in {\overline{W}}$ given by Theorem 2.5 in its form, not the solution $\sqrt{ f_0 }$ to the BCS-Bogoliubov gap equation. Suppose that the fixed point $f_0$ is an element of the subset *W*. It then follows immediately from Theorem 2.5 that $f_0 \in C^2(D)$. Hence the thermodynamic potential $\Omega$ with the fixed point $f_0$ satisfies all the conditions in the operator-theoretical definition of the second-order phase transition (see^2^[Definition 1.10]). We thus apply a proof similar to that of^2^[Theorem 2.4] to have Theorem 2.8.

Suppose that the fixed point $f_0$ is an accumulating point of the subset *W*. We then replace the fixed point $f_0 \in {\overline{W}} \setminus W$ in the form of the thermodynamic potential $\Omega$ by a suitably chosen element of $f \in W$ since the fixed point $f_0$ is an accumulating point of the subset *W*. Thanks to Theorem 2.5, we find that the suitably chosen element *f* is in $C^2(D)$. Then we can differentiate the suitably chosen element *f* with respect to the temperature *T* twice. Therefore, once we replace the fixed point $f_0 \in {\overline{W}} \setminus W$ in the form of the thermodynamic potential $\Omega$ by a suitably chosen element of $f \in W$, we can again show that the thermodynamic potential $\Omega$ with this $f \in W$ satisfies all the conditions in the operator-theoretical definition of the second-order phase transition. We can again apply a proof similar to that of^2^[Theorem 2.4] to have Theorem 2.8. This proves Theorem 2.8.
